# Simultaneous removal of tetracycline and Cu(ii) by adsorption and coadsorption using oxidized activated carbon

**DOI:** 10.1039/c7ra12402c

**Published:** 2018-01-08

**Authors:** Qingdong Qin, Xian Wu, Liwei Chen, Zhongshuai Jiang, Yan Xu

**Affiliations:** School of Civil Engineering, Southeast University Nanjing 210096 China xuxucalmm@seu.edu.cn +86 25 83790757 +86 25 83790757; College of Biology and the Environment, Nanjing Forestry University Nanjing 210037 China

## Abstract

Co-contamination of antibiotics and heavy metals prevails in the environment. To overcome the obstacle of low metal uptake on activated carbon and to achieve simultaneous removal of tetracycline (TC) and Cu(ii) from water, coconut shell based granular activated carbon (GAC) treated with nitric acid was utilized. GAC property characterization showed that oxidation treatment distinctly decreased the surface area of GAC and significantly increased the content of oxygen containing functional groups. The oxidized GAC exhibited greater adsorption capacity for individual TC and Cu(ii). Kinetics studies demonstrated that although the overall removal rate of coexisting TC and Cu(ii) decreased, the ultimate removal efficiency was further enhanced in the binary system. The adsorption isotherms were well described by Langmuir and Freundlich models. Moreover, the maximum adsorption capacities of coexisting TC and Cu(ii) with oxidized GAC kept increasing within a pH range of 3.0–6.0, indicating an electrostatic repulsion mechanism as well as a competition for adsorption sites. Fourier transform infrared spectroscopy (FTIR) and X-ray photoelectron spectroscopy (XPS) analysis revealed that the enhanced removal of TC and Cu(ii) was very likely as a result of coadsorption by forming TC–Cu(ii) complexes bridging between the adsorbate and the adsorbent.

## Abstract 2

The enhanced coadsorption of TC and Cu(ii) was likely due to the formation of a TC–Cu(ii) complex bridging.

## Graphical abstract

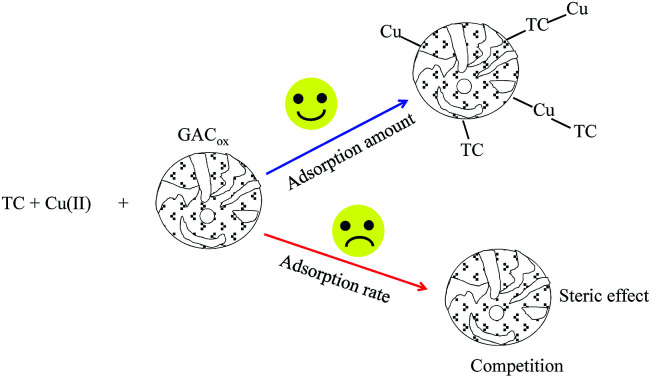

## 1. Introduction

Tetracycline antibiotics (TCs), such as tetracycline (TC), oxytetracycline (OTC), and chlorotetracycline (CTC) are widely used in livestock feeding operations and in disease control for humans and animals.^1^ It is reported that only a small portion of TCs is used in metabolism, 50–80% of the parental compounds are excreted through the feces and urine. As a result, TC residues are frequently detected in aquatic environments.^2^ Moreover, heavy metals have been commonly used in various chemical industries such as plating, mining and smelting, electroplating industries and petroleum refining.^3,4^ Also, natural water is polluted by heavy metals along with the discharge of industrial wastewater. Therefore, the coexistence of TCs and heavy metals is observed in the environment.^5–7^ This has led to great concern due to their increased stability and toxicity.^6,8–10^ For example, a long-term exposure to antibiotics and/or heavy metals causes the occurrence and spread of resistance genes.^8^ Most recently, a significant increase in TC resistance was found in the presence of environmentally relevant levels of Cu.^11^ Thus, the treatment of polluted aqueous effluents for simultaneous removal of TCs and heavy metals is crucial for water pollution control and ecological risk management.

To date, a variety of processes or methods have been widely employed for the removal of antibiotics or heavy metals from wastewater.^2,3^ Among the treatment options applied, considerable attentions have been paid to adsorption technology as an effective and simple separation process. Many materials are used as adsorbents, such as carbon adsorbents, clay and minerals, metal oxides and chitosan.^2,3,12^ As a most commonly used adsorbent, activated carbon is expected to have a high capacity for the removal of TCs due to its high specific surface area, abundant pore structures, and strong interactions. However, it has a relatively low adsorption capacity for the removal of heavy metals. The possible reason is the limited numbers of targeted functional groups such as –COOH, –OH, –NH_2_, –SH and –SO_3_H on the surface of activated carbon.^13–15^ Therefore, to improve the adsorption capacity for heavy metals, modification of activated carbon has been proposed.^16^

It has been shown that heavy metals adsorption by activated carbon greatly relies upon surface acidity and special surface functionality, where the removal mechanisms may comprise of electrostatic interaction, ion exchange and coordination to functional groups.^17,18^ A common technique to improve heavy metals adsorption is through chemical oxidation, which is able to introduce a variety of acidic surface functional groups on the surface of activated carbon.^19^ A variety of oxidizing agents such as HNO_3_, H_2_O_2_, (NH_4_)_2_S_2_O_8_, KMnO_4_, and NaOCl have been widely utilized.^20,21^ Among them, HNO_3_ is the most frequently used one, as its oxidizing specifications can be easily controlled by concentration and temperature. During oxidation, surface characteristics of activated carbon are altered due to the introduction of new functional groups such as carboxylic, phenolics, lactones and carbonyl, which can ultimately increase the adsorption capacity for heavy metal ions.^22^

So far not much is known about the interactions between TCs and heavy metals in binary system and the literature on adsorption process mainly focus on mono-component solutions.^2,3^ The presence of multiple pollutants in the same solution may significantly affect the removal performance of the adsorbent.^6,23,24^ The interaction between TCs and adsorbents may vary in the presence of heavy metals as well as the presence of TCs may change the behavior of heavy metals toward adsorbent. It has been reported that heavy metal ions and antibiotics could form ternary surface complexes, either through the adsorption of metal–ligand aqueous complexes by electrostatic forces (outer–sphere complexes), or through the formation of inner sphere structures.^6,23,25^ However, to the best knowledge of the authors, the shifts of the adsorption behaviors of TCs and heavy metals on granular activated carbon (GAC) remain unknown.

In this work, we chose TC and Cu(ii) as common representatives of antibiotics and heavy metals, respectively. The ultimate goal of the present study was to simultaneously remove TC and Cu(ii) from water. To achieve this objective, GAC was oxidized by nitric acid, and the influences of several operating parameters such as contact time, initial contaminant concentration and pH on the removal of TC and Cu(ii) were comprehensively investigated. Fourier transform infrared spectroscopy (FTIR) and X-ray photoelectron spectroscopy (XPS) of oxidized GAC before and after adsorption were also performed to identify the possible adsorption mechanisms. The improved performance of GAC in uptaking TC and Cu(ii) and the adsorption and coadsorption mechanisms can provide more insights of complex pollution treatment in aquatic systems.

## 2. Materials and methods

### 2.1. Materials

**Fig. 1:**
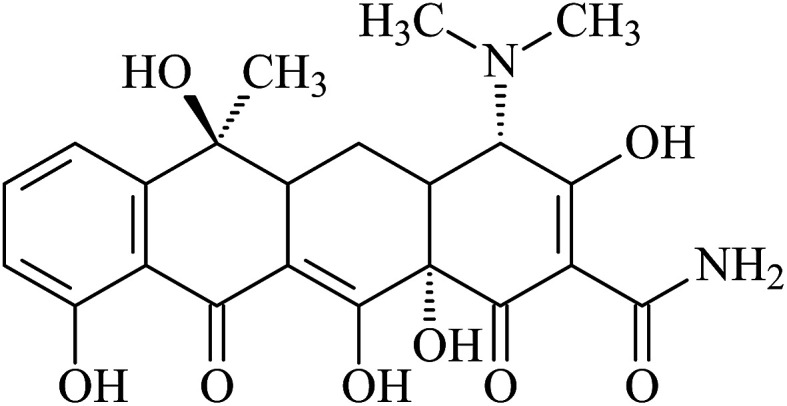
Structure of TC.

**Fig. 2:**
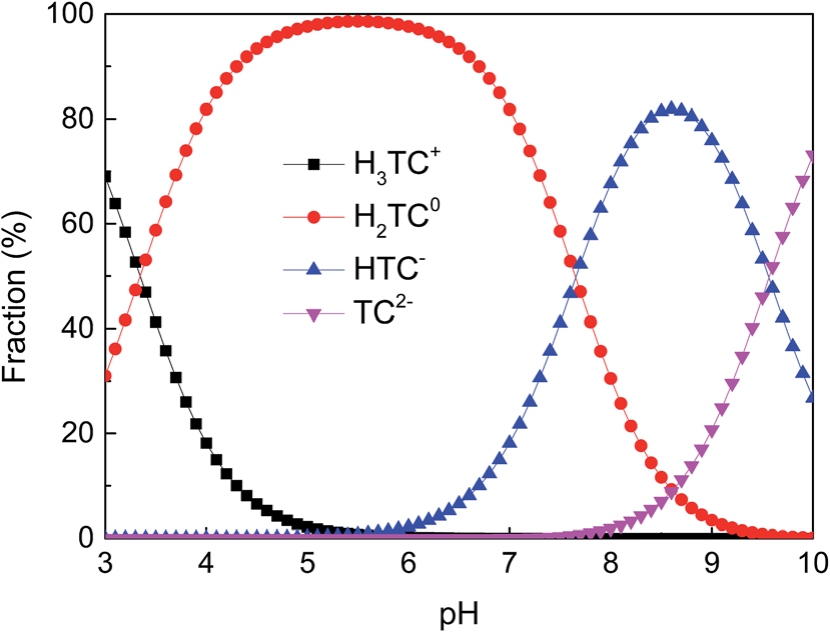
Distribution of TC species as a function of pH.

Tetracycline hydrochloride (TC, 95%, w/w) was purchased from Sigma-Aldrich and its molecular structure is shown in Fig. 1. It has multiple ionizable functional groups, including a dimethylammonium, a tricarbonylamide group and a phenolic diketone group, which exists three p*K*_a_ values of 3.3, 7.7 and 9.7.^26^ Therefore, due to protonation or deprotonation reactions, it shows different ionic species in different pH values, as depicted in Fig. 2.

All other chemicals (analytical grade) used in the study were purchased from Sigma-Aldrich and used without further purification. All solutions were prepared using 18 MΩ deionized H_2_O at neutral pH (Millipore, USA).

Coconut shell based granular activated carbon (GAC) kindly provided by Osaka Gas Chemicals Japan was used in this study. The particle size of GAC was 0.4–0.8 mm. The original GAC was washed by deionized water to an invariable pH value and desiccated at 378 K for 24 h. The oxidation of GAC was carried out as follows: 100 mL of a concentrated nitric acid solution (10 mol L^−1^) was introduced into a 250 mL flask and heated to 363 K using a constant temperature thermal bath device with condenser. Then 5 g of original GAC was immersed into the boiling nitric acid for 12 h. The modified GAC was washed with deionized water until to neutral pH and dried at 378 K for 24 h. Finally, it was designated as GAC_ox_.

### 2.2. Characterization

Prior to characterization, GAC and GAC_ox_ were dried at 105 °C for 24 h to remove the adsorbed moisture and were kept sealed under dry air in a desiccator. The surface acidic functional groups and acidic/basic sites were determined by Boehm titrations as described previously.^27^ The pH of the point of zero charge (pH_pzc_) of samples was established using a method suggested by Noh and Schwarz.^28^ Nitrogen adsorption–desorption isotherms were measured at 77 K on a Micromeritics ASAP 2020 sorptometer following the manufacture's introduction. Prior to measurement, the samples were outgassed for 16 h at 110 °C and 10^−6^ Torr. FTIR spectra were performed on the Spectrum One spectrometer from 400 to 4000 cm^−1^ by dried KBr pellet. XPS of the above mentioned samples were recorded on a spectrometer (Perkin-Elmer PHI-5300/ESCA, USA) with an Al Kα X-ray source. All the binding energies were referenced to the neutral C 1s peak at 284.6 eV to compensate for the surface charging effects. The XPS results were collected as binding energy forms and fitted using a curve-fitting program following the data analysis guide of XPSPEAK41 software.

### 2.3. Adsorption experiments

The effect of contact time on TC and Cu(ii) adsorption onto adsorbent was examined at pH 4.0. The adsorption experiments were carried out by mixing 0.1 g of adsorbent with a 250 mL aqueous solution in a 500 mL stirred flask at a temperature of 25 °C. Samples were periodically withdrawn to determine the residual concentrations of TC and Cu(ii) in the solution. The volume of each sample was 1 mL, and the total variation of the solution volume due to sampling was less than 6.0%.

The adsorption percentages of TC and Cu(ii) were calculated by the differences of initial and final concentrations using the equation expressed as follow:1
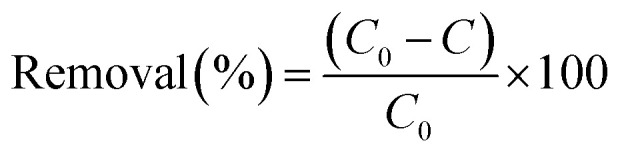
where *C*_0_ (mg L^−1^) is the initial concentration of adsorbate in solution, *C* (mg L^−1^) is the concentration of adsorbate in solution at time *t*.

The adsorption isotherm experiments of TC and Cu(ii) were performed using a batch experiment. In brief, 0.02 g of adsorbent was placed in a 100 mL flask, into which 50 mL of adsorbate solution with varying initial concentrations were added. The experiments were performed in a temperature-controlled water bath shaker for eight days (time to reach equilibrium) at a mixing speed of 180 rpm. When the equilibrium was thought to be established, the solutions were filtered and analyzed for the remaining concentration of adsorbate. Solid-phase adsorbate concentrations at equilibrium, *q*_e_ (mg g^−1^), were calculated according to eqn (2):2
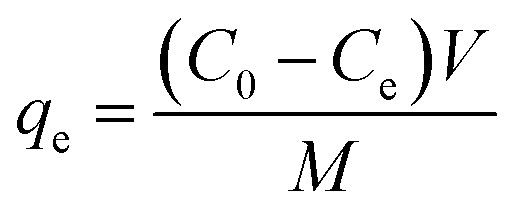
where *C*_e_ (mg L^−1^) is the equilibrium aqueous-phase concentration of adsorbate; *V* (L) is the volume of the aqueous solution; *M* (g) is the mass of dry adsorbent used in the experiments.

The effect of solution pH on adsorbate adsorption was investigated according to the following procedure. In brief, 0.02 g of GAC_ox_ was added to a series of 100 mL flasks each containing 50 mL of adsorbate solution. The solution pH was adjusted to a pH range from 3.0 to 6.0 using 0.1 mol L^−1^ HCl or 0.1 mol L^−1^ NaOH solutions. NaNO_3_ (0.01 mol L^−1^) was used to keep constant ionic strength. The flasks were then sealed and placed in the shaker at 25 °C with a speed of 150 rpm for eight days. Unless stated otherwise, all experiments were performed in duplicate.

### 2.4. Analytical method

Aqueous TC concentration was determined by a reversed phase High Performance Liquid Chromatography (HPLC) (Waters, USA) equipped with UV-visible detection at the wavelength of 360 nm, using a symmetry C18 column (4.6 × 150 mm, 5 μm spheres, Waters). The injection volume was 20 μL and the mobile phase was a mixture of methanol–water containing 0.01 mol L^−1^ oxalic acid at a flow rate of 1 mL min^−1^. Due to the presence of relatively high concentration of oxalic acid, Cu(ii) could not affect the UV absorption of TC. Moreover, it should be pointed out that no apparent peaks were detected in the HPLC spectra for potential degraded/transformed products of TC. The residual concentration of Cu(ii) in the solution was determined by inductively coupled plasma mass spectrometry (ICP-MS) method.

## 3. Results and discussion

### 3.1. Characterization of GAC and GAC_ox_

The textural and chemical characteristics of the carbon samples are given in Table 1. The oxidation treatment significantly decreased the specific surface area of the original GAC and almost completely destroyed the pore structure, which can be attributed to erosion and blockage of pores by degradation products produced during chemical reaction.^16^ Similar results were also found by other researchers using concentrated nitric acid to modify GAC.^29^ The elemental composition obtained from the XPS analysis showed a significant increase in oxygen content between the original and modified GAC. This is directly related to the increase in oxygen containing surface groups that was confirmed by acid/base titration. As a result, the pH_pzc_ value of GAC changed from 7.4 to less than 5.8, which is favored for adsorption of heavy metals under neutral pH.

**Table 1:** Textural and physicochemical properties of the GACs

GACs	*S*_BET_ (m^2^ g^−1^)	XPS (atom based) (%)			Acidic groups (mmol g^−1^)	Basic groups (mmol g^−1^)	pH_pzc_	Micropore area (m^2^ g^−1^)	External surface area (m^2^ g^−1^)
		C	N	O					
GAC	1392	95.19	0.58	4.23	0.25	0.39	7.4	930	461
GAC_ox_	48	80.76	1.62	17.61	1.07	0.82	5.8	40	8

### 3.2. Adsorption study

**Fig. 3:**
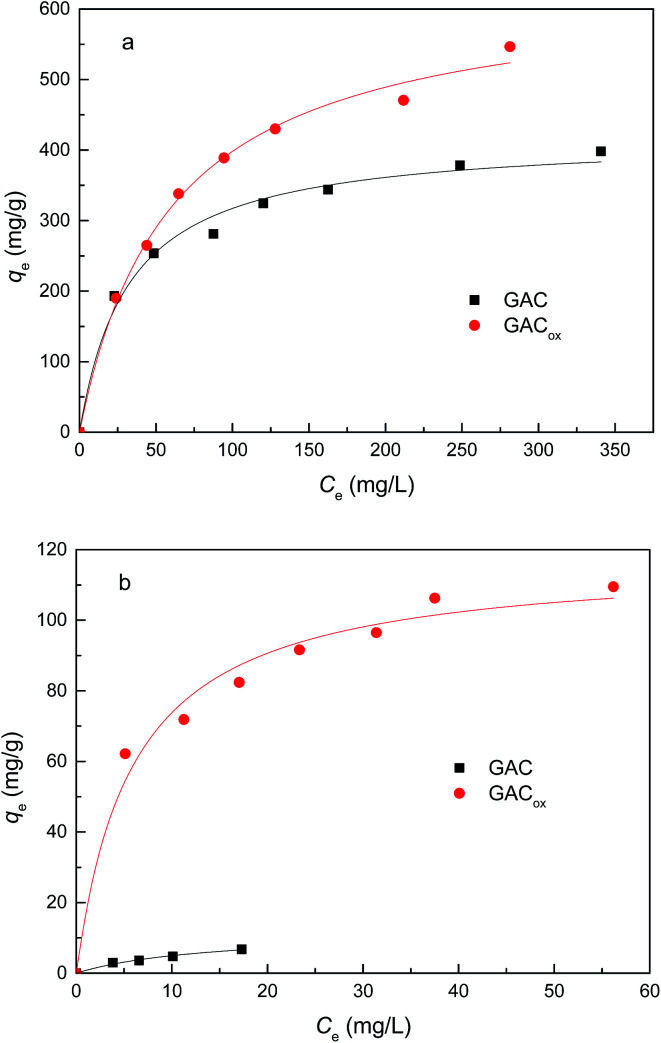
Adsorption of TC (a) and Cu(ii) (b) by GACs (adsorbent dosage = 0.4 g L^−1^, *T* = 25 °C, pH = 4.0).

Adsorption of individual TC and Cu(ii) on GAC with and without oxidization is shown in Fig. 3. It is observed that the amount of the TC or Cu(ii) adsorbed by GAC_ox_ was much greater than that of the raw GAC. Notably, the amount of the Cu(ii) adsorbed by GAC_ox_ was approximately one order of magnitude higher than that of raw GAC, which was mainly due to the increased content of acidic surface functional groups on the surface of GAC after oxidization. It was proposed that more content of acidic surface functional groups could reduce the pH_pzc_ (Table 1).^16^ At experimental pH 4.0, the surface of raw GAC was expected to be dominantly positive-charged, which was not conducive to Cu(ii) adsorption due to electrostatic repulsion. Although the surface of GAC_ox_ was also positive-charged, the content of acidic surface functional groups in GAC_ox_ was significantly higher than that in GAC, which favored Cu(ii) adsorption. Similar results were also observed by other researchers using oxidized activated carbon to remove heave metals.^14^ Therefore, GAC_ox_ was used in the following adsorption experiment.

#### 3.2.1. Effect of contact time

**Fig. 4:**
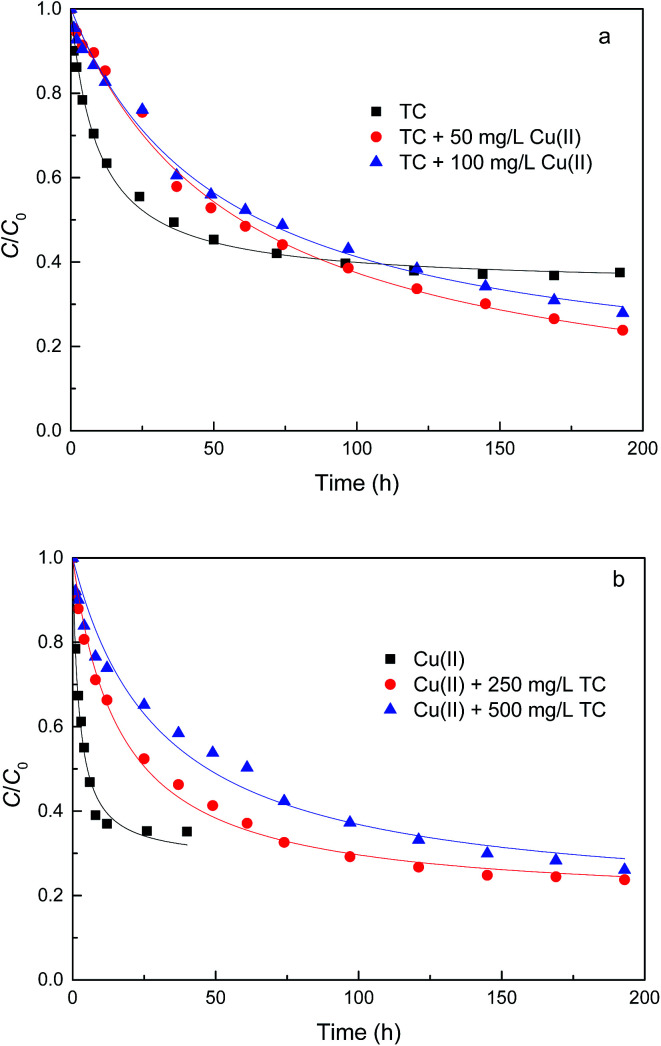
Effect of contact time on TC (a) and Cu(ii) (b) removal by GAC_ox_ (adsorbent dosage = 0.4 g L^−1^, *T* = 25 °C, pH = 4.0).

The removal of TC and Cu(ii) by GAC_ox_ varying with contact time is illustrated in Fig. 4. For TC adsorption (Fig. 4a), in single system, the equilibrium was reached after 120 h for TC adsorption and the removal of TC (initial concentration 250 mg L^−1^) at equilibrium was 62.1%. In binary system, where Cu(ii) was present in the solution, the equilibrium was reached after around 200 h. The removal of TC (initial concentration 250 mg L^−1^) in the presence of 50 mg L^−1^ Cu(ii) and 100 mg L^−1^ Cu(ii) was found to be 76.2% and 72.1%, respectively. The coexisting Cu(ii) even enhanced the overall adsorption of TC, but with a much longer equilibrium time. For Cu(ii) adsorption (Fig. 4b), the equilibrium time in single system was about 12 h and the removal of Cu(ii) (initial concentration 50 mg L^−1^) at equilibrium was 63.0%. When TC was coexisted in the solution, the equilibrium was reached after around 200 h. The removal of Cu(ii) (initial concentration 50 mg L^−1^) in the presence of 250 mg L^−1^ TC and 500 mg L^−1^ TC was 76.3% and 73.9%, respectively. In other words, the presence of TC favored the overall removal of Cu(ii) but with a much slower uptake rate. These results indicate that the coexistence of TC and Cu(ii) could significantly increase mass transfer resistances. On the other side, the synergic effect of TC and Cu(ii) leads to an increasing number of available adsorption sites on GAC_ox_.

In order to estimate the rate of TC and Cu(ii) adsorption on GAC_ox_, adsorption rate constants were determined by fitting the experimental adsorption kinetics data to first-order and second-order kinetic models, respectively.^30,31^

The pseudo-first-order kinetic model is given as follows:

3 *q*_*t*_ = *q*_e_(1 − e^−*k*_1_*t*^)

The above equation can also be expressed in terms of *C* by using the mass balance equation at time *t* as follows:4
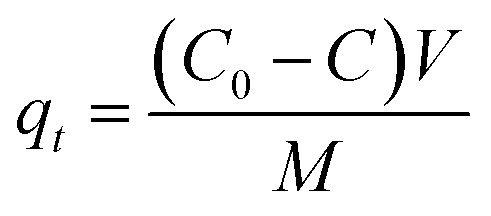
5
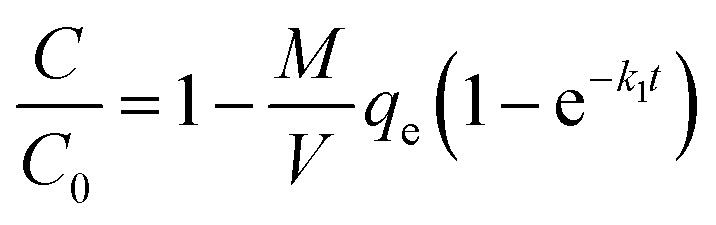


The pseudo-second-order kinetic model is expressed by the following equation:6
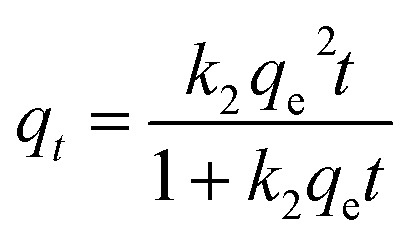


This equation can be also formulated in terms of *C*, yielding the following equation:7
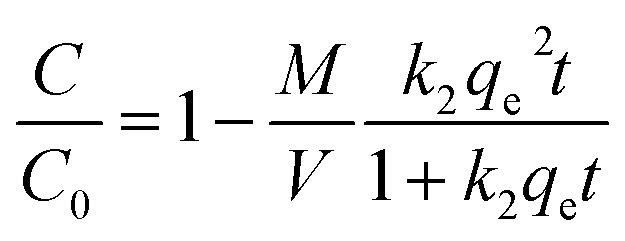
where *q*_*t*_ is the amount of adsorbate adsorbed on GAC_ox_ at time *t* (h), *k*_1_ is the rate constant of pseudo-first-order adsorption (h^−1^), *k*_2_ is the rate constant of the pseudo-second-order model (g mg^−1^ × h).

**Fig. 5:**
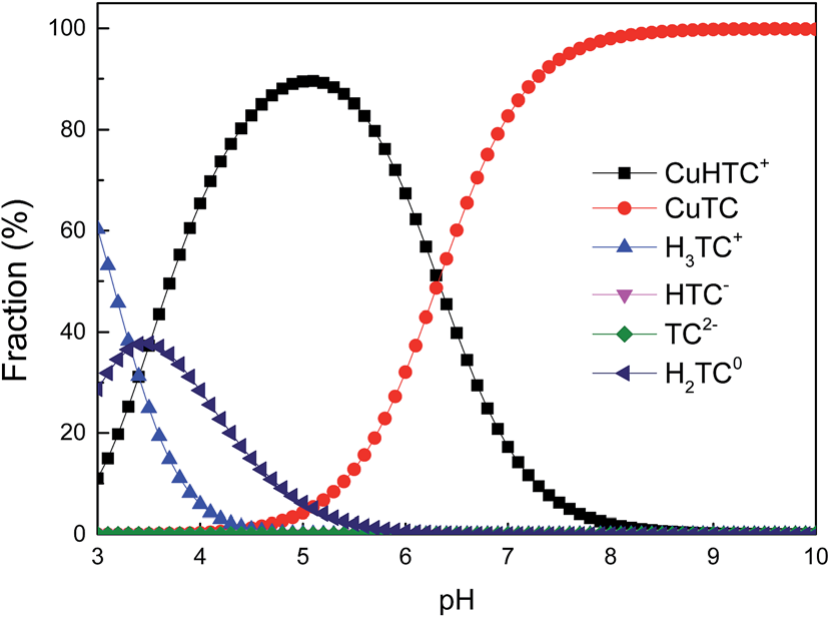
Speciation distributions of TC complexation with Cu(ii) as a function of pH (TC = 250 mg L^−1^, Cu(ii) = 50 mg L^−1^).

The results from fitting experimental data with pseudo-first and pseudo-second-order models are presented in Table 2. It is clear to see that adsorption rate could be fitted well using both two kinetic models (evidenced from the correlation coefficients, >0.970). The *k*_1_ and *k*_2_ values decreased significantly in the presence of TC or Cu(ii), indicating the increase of resistance to mass transfer. The possible reason might be competition adsorption and steric effect. Given that TC and Cu(ii) can be both adsorbed by GAC_ox_, they may compete with each other for space and/or sites on GAC_ox_, resulting in a decrease of adsorption rate. Moreover, TC and Cu(ii) could form complexes in the binary system. The complexation constants (log *K*) of TC with Cu(ii) were −0.6 and −7.3 and the speciation distributions of TC complexation with Cu(ii) are shown in Fig. 5.^26^ It can been seen that approximately 65.4% of the TC forms CuHTC^+^ with Cu(ii) at pH 4.0, which leads to the increasing of molecular size and forms pore blockage and finally reduces the adsorption rate. These results provide more insights in the design of an adsorption column, as the adsorption rate in the binary system is significantly slower than that in the single system.

**Table 2:** Adsorption rate parameters for the pseudo-first and pseudo-second-order models

Adsorbate	Coexisting compound	Pseudo-first-order model		Pseudo-second-order model	
		*k*_1_ (h^−1^)	*R* ^2^	*k*_2_ (g mg^−1^ × h)	*R* ^2^
TC	No	0.058	0.979	0.065	0.994
	50 mg L^−1^ Cu(ii)	0.018	0.994	0.007	0.993
	100 mg L^−1^ Cu(ii)	0.017	0.992	0.008	0.992
Cu(ii)	No	0.312	0.993	0.246	0.986
	250 mg L^−1^ TC	0.035	0.984	0.030	0.993
	500 mg L^−1^ TC	0.019	0.980	0.016	0.974

#### 3.2.2. Adsorption isotherms

**Fig. 6:**
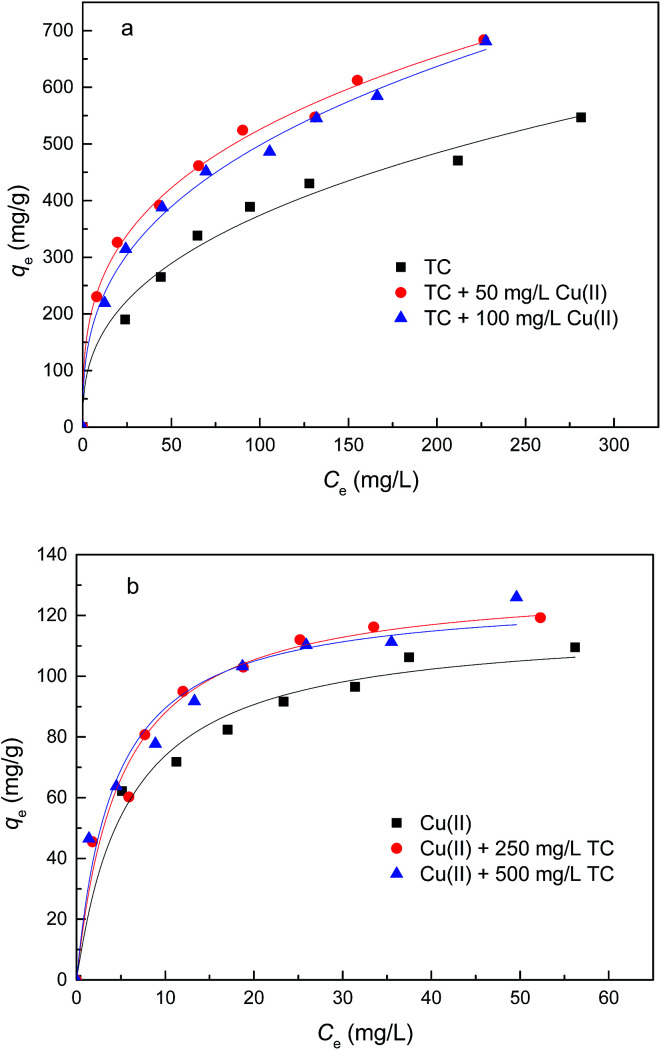
Adsorption isotherms of TC (a) and Cu(ii) (b) on GAC_ox_ (adsorbent dosage = 0.4 g L^−1^, *T* = 25 °C, pH = 4.0).

Adsorption isotherms of TC and Cu(ii) onto GAC_ox_ at temperature of 25 °C are plotted in Fig. 6. As seen in Fig. 6a, the amount of TC adsorbed on GAC_ox_ in the absence of Cu(ii) increased from 190.0 to 546.7 mg g^−1^ when the initial TC concentration increased from 100 to 500 mg L^−1^. The adsorption amount of TC in the presence of Cu(ii) was also investigated, and the results reveal that the coexisting Cu(ii) increases the adsorption capacity for TC onto GAC_ox_. Similarly, the adsorption of Cu(ii) was also enhanced in the presence of TC (Fig. 6b).

In order to describe the adsorption isotherms, two most important isotherms were used in this study, the Langmuir and Freundlich isotherms:8
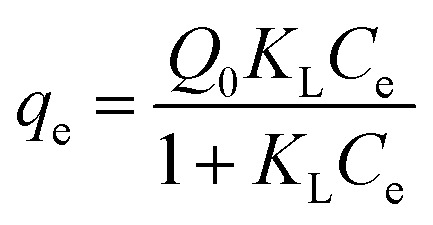

9 *q*_e_ = *K*_F_*C*_e_^1/*n*^

where *q*_e_ (mg g^−1^) is the amount of adsorbate adsorbed per gram of GAC_ox_ at equilibrium; *C*_e_ (mg L^−1^) is the equilibrium concentration of adsorbate in solution; *Q*_0_ (mg g^−1^) is the maximum monolayer adsorption capacity; *K*_L_ (L mg^−1^) is the constant related to the free energy of adsorption; *K*_F_ is a Freundlich isotherm constant for the system and the slope 1/*n*, ranging between 0 and 1, is an indicative of the degree of nonlinearity between solution concentration and adsorption. The isotherm parameters obtained from the fitting curves by Langmuir and Freundlich models are given in Table 3. It is clear to see that adsorption isotherms of TC and Cu(ii) can be fitted well using Langmuir and Freundlich models. To facilitate comparison, the maximum adsorption capacities were discussed according to results obtained from the Langmuir model. The maximum monolayer adsorption capacity (*Q*_0_) of TC increased from 634.0 mg g^−1^ to 691.1 mg g^−1^, when the concentration of Cu(ii) increased from 0 to 50 mg L^−1^. Similar trends were also observed in Cu(ii) adsorption. The enhanced adsorption of TC and Cu(ii) might be ascribed to the strong complex formation between TC and Cu(ii). As TC and Cu(ii) can be both adsorbed by GAC_ox_, the TC and Cu(ii) could act as a bridge between the adsorbate and the adsorbent, leading to the increase of TC and Cu(ii) adsorption. Moreover, when the concentration of TC or Cu(ii) further increased, the *Q*_0_ did not change significantly. The possible reason may be the competition adsorption and/or pore blockage at a higher TC or Cu(ii) concentration.

**Table 3:** Parameters of adsorption isotherms of TC and Cu(ii) on GAC_ox_

Adsorbate	Coexisting compound	Langmuir model			Freundlich model		
		*Q*_0_ (mg g^−1^)	*K*_L_ (L mg^−1^)	*R* ^2^	*K*_F_	1/*n*	*R* ^2^
TC	No	634.0	0.017	0.993	67.4	0.37	0.961
	50 mg L^−1^ Cu(ii)	691.1	0.040	0.956	122.9	0.32	0.991
	100 mg L^−1^ Cu(ii)	714.8	0.028	0.970	97.0	0.36	0.988
Cu(ii)	No	117.4	0.169	0.980	40.4	0.25	0.971
	250 mg L^−1^ TC	131.4	0.203	0.977	43.9	0.27	0.906
	500 mg L^−1^ TC	126.8	0.240	0.963	43.8	0.27	0.979

To compare the performance of GAC_ox_ with other adsorbents, the adsorption capacities for organic pollutants and heavy metals found in binary system are summarized in Table 4. Comparative values of *Q*_0_ clearly suggested that GAC_ox_ exhibited excellent adsorption for TC and relatively high adsorption for Cu(ii). These results indicate that GAC_ox_ can serve as a potential adsorbent for the simultaneous removal of TC and Cu(ii) from contaminated water.

**Table 4:** Comparison of adsorption capacities of other adsorbents for simultaneous removal of organic pollutants and heavy metals in binary system^a^

Adsorbents	pH	*T* (°C)	*Q*_0_ (mg g^−1^)		References
			Organic pollutants	Heavy metals	
GAC_ox_	4.0	25	714.8 (TC)	126.8 (Cu(ii))	This work
Chitosan	5.0	25	41.4 (TC)	95.1 (Cu(ii))	32
Chelating resin	5.0	25	107.1 (TC)	128.4 (Cu(ii))	33
Activated carbon	6.0	25	442.5 (CIP)	4.7 (Ni(ii))	34
Bifunctional resin	5.0	20	230.1 (TC)	38.2 (Cu(ii))	25
Fe–*N*,*N*-SBA15	5.5	25	49.9 (TC)	36.0 (Cu(ii))	24
MMIC-Fe(iii)	8.0	25	516.3 (TC)	194.3 (Cd(ii))	12

a Abbreviations in this table: CIP, ciprofloxacin.

#### 3.2.3. Effect of pH

**Fig. 7:**
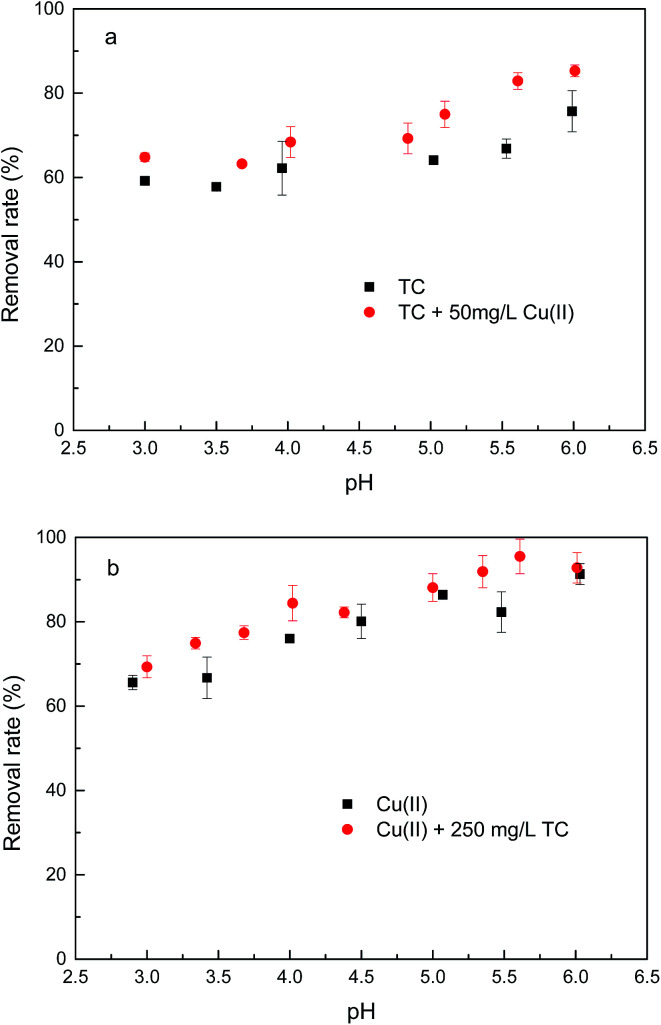
Effect of pH on TC (a) and Cu(ii) (b) by GAC_ox_ (adsorbent dosage = 0.4 g L^−1^, *T* = 25 °C).

The pH values of solution can affect surface charges of GAC_ox_, as well as the degree of the ionization of the TC. The speciation of TC molecules can exist as cation species H_3_TC^+^ (pH < 3.3), zwitterion species H_2_TC^0^ (3.3 < pH < 7.7) and anion species HTC^−^ and TC^2−^ (pH > 7.7). The pH_pzc_ of GAC_ox_ was 5.8. The surface of GAC_ox_ was positively charged at pH < pH_pzc_, whereas the surface of GAC_ox_ was negatively charged at pH > pH_pzc_. Fig. 7 shows the influence of solution pH on the removal of TC and Cu(ii) by GAC_ox_. Obviously, solution pH is a crucial factor to affect the extent of TC and Cu(ii) adsorption onto GAC_ox_. The removal of TC and Cu(ii) was enhanced in the binary system ranged from pH 3.0 to 6.0. Similar observation was also found in the coadsorption of TC and Cu(ii) on other adsorbents at pH < 6.0.^25,26^

As seen in Fig. 7a, the removal of TC in the absence of Cu(ii) went up with increasing pH up to 6.0. At pH 6.0, the removal of TC achieved 75.7%. At the pH below 6.0, the low removal efficiencies were mainly due to a repulsive force prevailing between the cationic species of TC in solution and the positively charged surface of GAC_ox_, and/or a competition between H^+^ ions and the cationic species of TC for binding sites on the surface of GAC_ox_. When the pH increased from 3.0 to 6.0, most of TC existed as zwitterionic species (Fig. 2). Thus, the adsorption of TC on GAC_ox_ increased along with the decreased electrostatic repulsion between TC and the positively charged surface of GAC_ox_. When Cu(ii) was coexisted in the solution, the removal of TC slightly increased in the pH range of 3.0–6.0. The removal of TC in the presence of Cu(ii) reached 85.3% at pH 6.0. Seen in Fig. 5, the predominant TC species is CuHTC^+^ at pH values between 4.0 and 6.0. Meanwhile, the surface of GAC_ox_ was positively charged at pH < 5.8. Therefore, it is expected that the removal of TC in the presence of Cu(ii) should be lower than that in the absence of Cu(ii) due to electrostatic repulsion. However, the observed removal of TC even slightly increased in the presence of Cu(ii). This phenomenon was possibly due to the complexation of TC with the adsorbed Cu(ii), that was, through formation of a GAC_ox_–Cu(ii)–TC ternary complex.^7^

As seen in Fig. 7b, the removal of Cu(ii) in the absence of TC with an increase of pH from 3.0 to 6.0 raised from 65.6% to 91.3%. At pH values below the onset of Cu(ii) precipitation, the variations in adsorbed amount with pH could be explained on the basis of the number of negatively charged sites on the surface of GAC_ox_. As pH increased, more and more H^+^ ions tended to leave the GAC_ox_ surface resulting in more negatively charged sites available, which favored Cu(ii) adsorption due to the reduced electrostatic repulsion. Hence, positively charged Cu(ii) ions will ion exchange and/or complex with oxygenated surface functional groups at pH values in the range 3.0–6.0. In the presence of TC, TC was found to favor Cu(ii) adsorption at pH < 6.0. The role of TC on Cu(ii) adsorption may incorporate several different mechanisms: (1) enhanced adsorption by forming ternary surface complexes, (2) reduced Cu(ii) adsorption due to competition between the surface ligands and the TC ligands for Cu(ii), and (3) competition between TC and Cu(ii) for surface sites.^6^ Therefore, the reason for the enhanced Cu(ii) adsorption was most likely due to Cu(ii) adsorption onto GAC_ox_ surface through complexation of Cu(ii) with the adsorbed TC to form a GAC_ox_–TC–Cu(ii) ternary complex. Additionally, in the pH range of 3.0–6.0, Cu(ii) can complex with H_2_TC^0^ to form CuHTC^+^ species (Fig. 5), which helps to decrease the number of positively charged Cu(ii) and reduce electrostatic repulsion between Cu(ii) and the positively charged surface of GAC_ox_ and ultimately enhances the Cu(ii) adsorption on GAC_ox_.

### 3.3. FTIR and XPS analysis

**Fig. 8:**
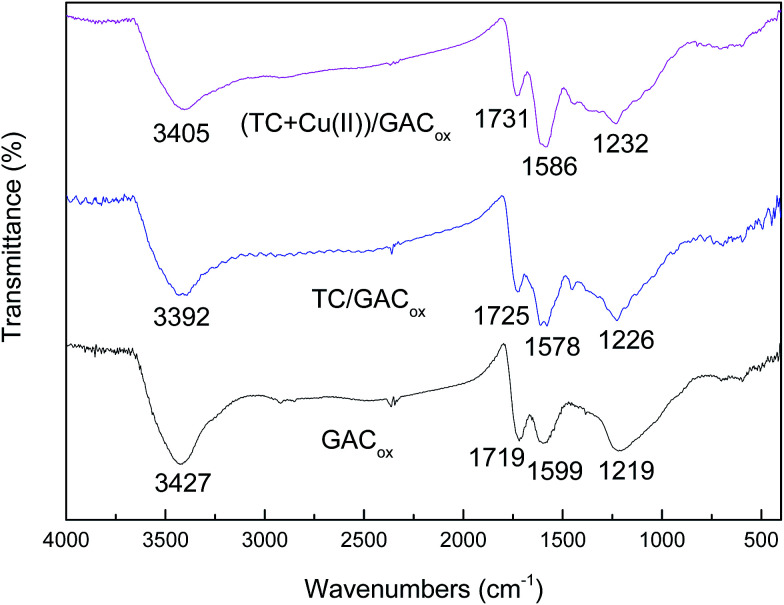
FTIR spectra of GAC_ox_ before and after adsorption.

The FTIR spectra of GAC_ox_ before and after adsorption are shown in Fig. 8. In the case of pure GAC_ox_, the spectrum exhibited the absorption bands at 3427, 1719, 1599 and 1219 cm^−1^. The broadened band around 3427 cm^−1^ can be assigned to the bending vibration of adsorbed molecular water and stretching vibration of hydroxyl group. The peak at 1719 cm^−1^ was attributed to the stretching vibration of C=O in ketones, lactones, and carboxyl groups.^35,36^ The peak at 1599 cm^−1^ was ascribed to aromatic ring stretching vibration.^35,36^ The broadened band around 1219 cm^−1^ was usually attributed to a C–O bond.^35^ It can be seen that the peaks at 1719, 1599 and 1219 cm^−1^ shifted after TC adsorption. This suggests that these groups participate in the formation of a coordination complex with TC. When TC and Cu(ii) were adsorbed on GAC_ox_ simultaneously, the observed FTIR spectra were different from those observed in the absence of Cu(ii), indicating that Cu(ii) adsorption can take place on the sites where TC was specifically adsorbed. It has been reported that the main mechanism of adsorption of TC by activated carbon including hydrogen bonding, electron donor–acceptor, and π–π dispersion interaction between the aromatic ring of three antibiotics and the delocalized π electrons present in GAC_ox_.^2^ The Cu(ii) adsorption is mainly due to the formation of metal surface complex with hydroxyl and carboxylic groups on the surface of GAC_ox_.^16,22^ Seen in Fig. 8, when TC and TC/Cu(ii) were adsorbed on GAC_ox_, the peak at 1599 cm^−1^ ascribed to aromatic ring stretching vibration was shifted to 1578 cm^−1^ and 1586 cm^−1^, respectively. These shifts indicate that TC–Cu(ii) complexes can be coadsorbed by GAC_ox_.

**Fig. 9:**
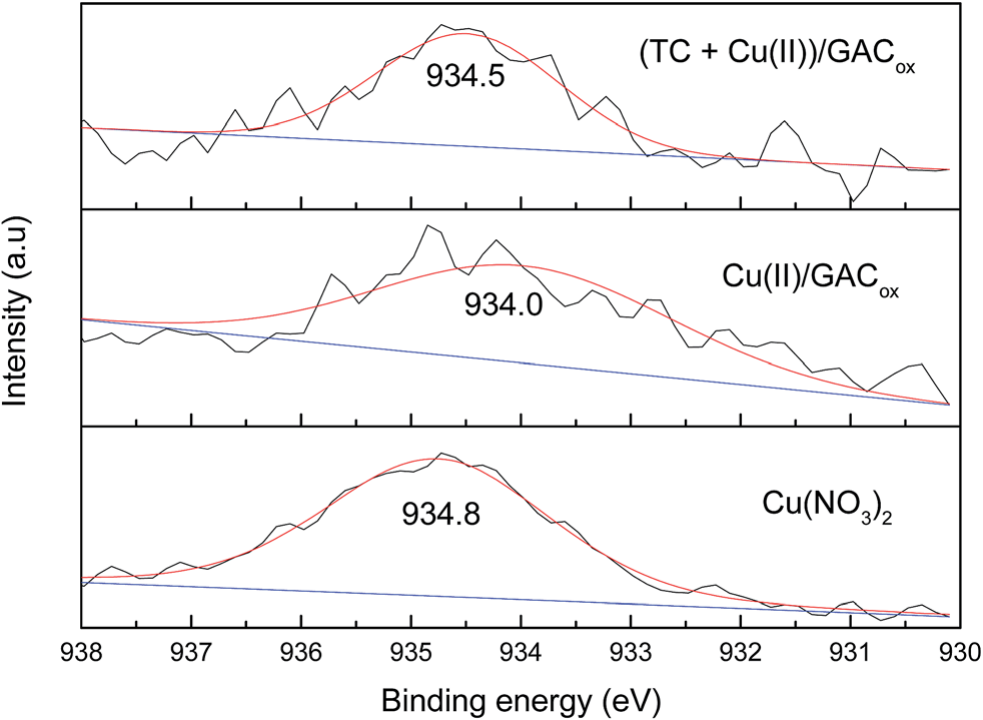
XPS spectra of GAC_ox_ before and after adsorption.

To gain further insight into the mechanism of adsorption and coadsorption onto GAC_ox_, high resolution XPS Cu 2p_2/3_ spectra were carefully analyzed (Fig. 9). The peak of binding energy for Cu(NO_3_)_2_ appeared at 934.8 eV (assigned to Cu 2p_2/3_). After adsorption by GAC_ox_, a remarkable shift to the low region (from 934.8 to 934.0 eV) was observed, indicating a specific interaction between Cu(ii) and GAC_ox_. Furthermore, when TC was coexisted, the peak changed to 934.5 eV, suggesting that TC could directly affect the binding energy of Cu(ii) due to complexation. These findings indicate that the enhanced adsorption of TC in the presence of Cu(ii) and the enhanced adsorption of Cu(ii) in the presence of TC both could be resulted from the formation of ternary complexes of GAC_ox_–Cu(ii)–TC and GAC_ox_–TC–Cu(ii) during adsorption.

## 4. Conclusions

The present study shows that the treatment by HNO_3_ significantly changed the physicochemical properties of GAC such as pH_pzc_, surface area and groups. Compared to raw GAC, GAC_ox_ was able to adsorb significantly higher amounts of Cu(ii) mainly due to the increased content of acidic surface functional groups on the surface of GAC_ox_. Kinetics studies demonstrated that the removal rate decreased by approximately 69% for TC and by 94% for Cu(ii) in the binary system. The maximum adsorption capacity was 634.0 mg g^−1^ for TC and was 117.4 mg g^−1^ for Cu(ii) in the single system. While, in binary system, the maximum adsorption capacity increased to 714.8 mg g^−1^ for TC and 131.4 mg g^−1^ for Cu(ii). Moreover, overall adsorption of coexisting TC and Cu(ii) by GAC_ox_ went up within a pH range of 3.0–6.0. Batch experiments and FTIR and XPS analyses revealed that the enhanced adsorption of coexisting TC and Cu(ii) might be due to the formation of TC–Cu(ii) complex bridging between the adsorbate and the adsorbent. Compared to other adsorbents, GAC_ox_ showed excellent adsorption for TC and relatively high adsorption for Cu(ii) in binary system. These results suggest the considerable potential for the oxidized GAC as an adsorbent for the simultaneous removal of TC and Cu(ii) from contaminated water.

## Conflicts of interest

There are no conflicts to declare.

## Supplementary Material

## References

[cit1] Sarmah A. K., Meyer M. T., Boxall A. B. A. (2006). Chemosphere.

[cit2] Ahmed M. B., Zhou J. L., Ngo H. H., Guo W. S. (2015). Sci. Total Environ..

[cit3] Uddin M. K. (2017). Chem. Eng. J..

[cit4] Ray P. Z., Shipley H. J. (2015). RSC Adv..

[cit5] Vystavna Y., Le Coustumer P., Huneau F. (2013). Environ. Monit. Assess..

[cit6] Wang Y. J., Jia D. A., Sun R. J., Zhu H. W., Zhou D. M. (2008). Environ. Sci. Technol..

[cit7] Jia D. A., Zhou D. M., Wang Y. J., Zhu H. W., Chen J. L. (2008). Geoderma.

[cit8] Zhang Y., Cai X. Y., Lang X. M., Qiao X. L., Li X. H., Chen J. W. (2012). Environ. Pollut..

[cit9] Tuckfield R. C., McArthur J. V. (2008). Microb. Ecol..

[cit10] Berg J., Thorsen M. K., Holm P. E., Jensen J., Nybroe O., Brandt K. K. (2010). Environ. Sci. Technol..

[cit11] Song J. X., Rensing C., Holm P. E., Virta M., Brandt K. K. (2017). Environ. Sci. Technol..

[cit12] Chen A. W., Shang C., Shao J. H., Lin Y. Q., Luo S., Zhang J. C., Huang H. L., Lei M., Zeng Q. R. (2017). Carbohydr. Polym..

[cit13] Li N., Ma X. L., Zha Q. F., Kim K., Chen Y. S., Song C. S. (2011). Carbon.

[cit14] Cho H. H., Wepasnick K., Smith B. A., Bangash F. K., Fairbrother D. H., Ball W. P. (2010). Langmuir.

[cit15] Xu L. J., Wang J. L. (2012). Appl. Catal., B.

[cit16] Rivera-Utrilla J., Sanchez-Polo M., Gomez-Serrano V., Alvarez P. M., Alvim-Ferraz M. C. M., Dias J. M. (2011). J. Hazard. Mater..

[cit17] Rivera-Utrilla J., Sanchez-Polo M. (2003). Water Res..

[cit18] Yin C. Y., Aroua M. K., Daud W. (2007). Sep. Purif. Technol..

[cit19] Strelko V., Malik D. J. (2002). J. Colloid Interface Sci..

[cit20] Pradhan B. K., Sandle N. K. (1999). Carbon.

[cit21] Daud W., Houshamnd A. H. (2010). J. Nat. Gas Chem..

[cit22] Chen J. P., Wu S. N. (2004). Langmuir.

[cit23] Pei Z. G., Shan X. Q., Kong J. J., Wen B., Owens G. (2010). Environ. Sci. Technol..

[cit24] Zhang Z. Y., Liu H. J., Wu L. Y., Lan H. C., Qu J.
H. (2015). Chemosphere.

[cit25] Ma Y., Zhou Q., Zhou S. C., Wang W., Jin J., Xie J. W., Li A. M., Shuang C. D. (2014). Chem. Eng. J..

[cit26] Zhao Y. P., Tan Y. Y., Guo Y., Gu X. Y., Wang X. R., Zhang Y. (2013). Environ. Pollut..

[cit27] Goertzen S. L., Theriault K. D., Oickle A. M., Tarasuk A. C., Andreas H. A. (2010). Carbon.

[cit28] Noh J. S., Schwarz J. A. (1989). J. Colloid Interface Sci..

[cit29] Liu G. F., Ma J., Li X. C., Qin Q. D. (2009). J. Hazard. Mater..

[cit30] Blanchard G., Maunaye M., Martin G. (1984). Water Res..

[cit31] Ocampo-Perez R., Leyua-Ramos R., Rivera-Utrilla J., Flores-Cano J. V., Sanchez-Polo M. (2015). Chem. Eng. Res. Des..

[cit32] Kang J., Liu H. J., Zheng Y. M., Qu J. H., Chen J. P. (2010). J. Colloid Interface Sci..

[cit33] Ling C., Liu F. Q., Xu C., Chen T. P., Li A. M. (2013). ACS Appl. Mater. Interfaces.

[cit34] Sun Y. Y., Yue Q. Y., Gao B. Y., Gao Y., Xu X., Li Q., Wang Y. (2014). J. Taiwan Inst. Chem. Eng..

[cit35] Guo Y. P., Rockstraw D. A. (2006). Carbon.

[cit36] Demiral H., Gungor C. (2016). J. Cleaner Prod..

